# Initial shoot regeneration in the selenium hyperaccumulator *Neptunia amplexicaulis* and in vitro test system for selenium tolerance and accumulation

**DOI:** 10.1007/s13659-025-00532-9

**Published:** 2025-08-04

**Authors:** Bennet Buhmann, Jeroen van der Woude, Traud Winkelmann, Antony van der Ent

**Affiliations:** 1 Institute of Plant Genetics, Section Reproduction and Development, Leibniz University Hannover, Hannover, Germany; 2 Laboratory of Genetics, Wageningen University and Research, Wageningen, The Netherlands

**Keywords:** Hyperaccumulator, *Neptunia amplexicaulis*, *Neptunia heliophila*, *Medicago truncatula*, Selenium, Selenate

## Abstract

The trace element selenium is essential for human nutrition but is distributed unevenly in soils worldwide with extensive selenium-deficient regions and selenium-enriched (seleniferous) areas. *Neptunia amplexicaulis* is one of the strongest selenium hyperaccumulator plants known and native to Australian seleniferous soils. Research in the genetic background of the selenium accumulation and tolerance mechanisms of this species lacks biotechnological and molecular tools for functional genetics. Therefore, this study aimed to develop a de novo shoot regeneration protocol for *N. amplexicaulis* and validate an selenium accumulation test system. Callus was induced on root and hypocotyl explants excised from 5-day old seedlings and cultured on an adjusted MS medium (SIM9) containing 4.5 µM Thidiazuron (TDZ) for two weeks in darkness. After this period, the TDZ concentration was reduced to 0.45 µM, and the explants were transferred to light conditions. In addition, seedlings of *N. amplexicaulis*, *N. heliophila* and *Medicago truncatula* were placed on vertical MS agar plates containing 1.5 mM (standard) or 0.1 mM (low) magnesium sulphate with 0, 30, 90 µM sodium selenate. Initial shoot differentiation was observed 6 weeks after culture initiation. This regeneration response was successfully repeated in a second experiment. The outgrow of the shoot buds into complete shoots was not yet achieved but requires additional media optimization. Additionally, spontaneous shoot regeneration from a root was observed, highlighting potential for further studies. In vitro grown seedlings demonstrated efficient, selective selenium uptake in *N. amplexicaulis* and identified *M. truncatula* as a secondary selenium accumulator with selenium concentrations of > 300 µg Se g^−1^ DM. This project presents the first protocol for inducing early stages of development of indirect shoot organogenesis in *N. amplexicaulis* from hypocotyl and root explants as prerequisite for genetic transformation, though completing the regeneration cycle remains challenging. *Neptunia amplexicaulis* hyperaccumulates selenium also under in vitro conditions.

**Graphical Abstract:**

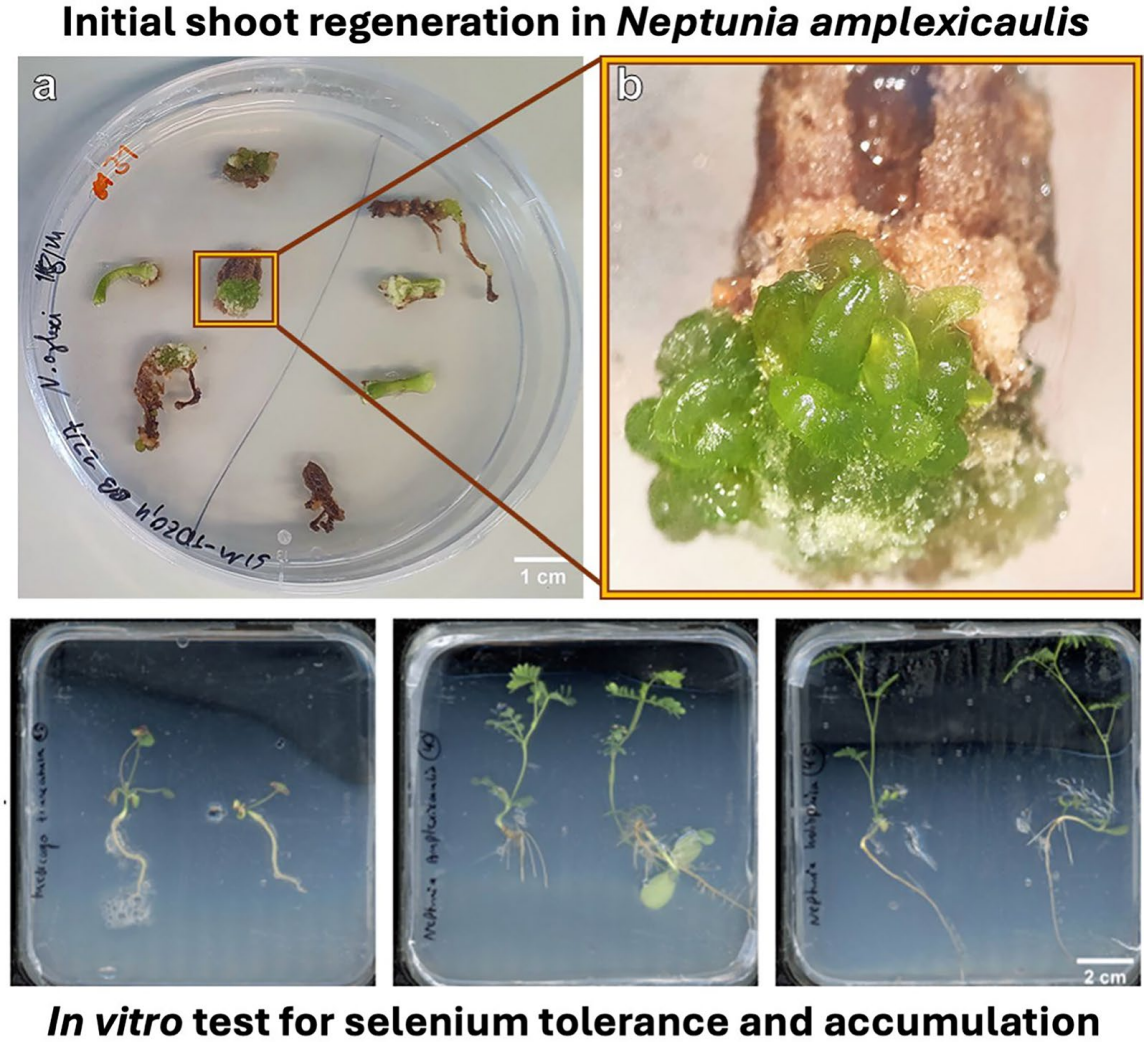

**Supplementary Information:**

The online version contains supplementary material available at 10.1007/s13659-025-00532-9.

## Introduction

Selenium (Se) is a trace element that is essential for human and animal nutrition [1]. The critical aspect of trace elements is that the metabolism requires only a small but regularly amount of 55–70 µg Se per day [2]. The geological distribution in the Earth's crust is heterogenic resulting in large Se deficient areas in East Europe, China and USA and Se enriched (‘seleniferous’) areas in USA and Australia [3, 4]. This disparity has led to a global Se deficiency approximately affecting one billion people [5]. Even though Se has not been recognized as an essential element for plants yet plants play a key role in human Se dietary as they are able to absorb and metabolize inorganic Se making Se appropriate for entering the human food chain [6]. Genetically based biofortification strategies are currently limited due low Se accumulation and tolerance and inefficient Se uptake in conventional crop species [7]. A strategy to overcome this bottleneck is to investigate existing Se-tolerant species, such as *Astragalus bisulcatus* or *Neptunia amplexicaulis*, and incorporate their traits into breeding programs of conventional crop species, potentially through transgenic integration [7]. Understanding the specific metabolic pathways and Se tolerance mechanisms of rare Se hyperaccumulator species is therefore of great interest [8].

*Neptunia amplexicaulis* Domin (Fabaceae) is a perennial shrub and belongs to the subfamily of Mimosaceae and occurs endemically on Se-enriched so called seleniferous soils in and around the Richmond District of Queensland, Australia in a semi-arid climate [9, 10]. In its natural environment *N. amplexicaulis* accumulates up to 3000–4000 µg Se g^−1^ but Se concentration can increase to 13,600 µg Se g^−1^ in young leaves and shoot tips under artificial greenhouse conditions [11]. *N. amplexicaulis* emerges as a model species for hyperaccumulation, shedding light on molecular mechanisms and evolutionary drivers through comprehensive studies including the closely related secondary accumulator *Neptunia heliophila*. So far, genetic tools have not been established for these species. Because of the chemical similarity between Se and sulfur (S), it was known quite early that Se is metabolized via the same pathways as S [12, 13]. In *N. amplexicaulis* it was shown that the highest concentrations of Se are found in young shoots and leaves, the taproot, vascular tissue and in the seedpods. The mechanism driving this distribution on the molecular, regulatory, and evolutionary level are unknown and cannot be explained based on the current knowledge of the assimilation pathway. Therefore, Se-sensing, transport, and assimilation are of special interest to be investigated in the context of Se hyperaccumulation. Most of the genes involved in Se assimilation show increased expression rates during a S deficiency in non-accumulators [14]. These expression patterns are also induced by higher selenate concentrations. That suggests that Se is perceived as a lack of S; conversely, plants are more tolerant to Se when they have a higher S supply [15]. Although non-accumulators only weakly regulate the Se:S ratio, with increasing Se supply, the ratio increases [16]. In a transcriptomic expression analysis of *S. pinnata* from Wang et al. [14], it has been shown that the expression of S assimilation genes, including for transporters, are constitutively highly expressed in hyperaccumulators.

The principles of Se assimilation in non-accumulator plants have been explored through gene knockout studies but remain underexplored in hyperaccumulator species. Genetic transformation techniques, allowing gene overexpression and knockouts, are well-established fundamental tools in plant research, offering insights into metabolic pathways and functional genetics [17]. Especially the discovery of Clustered Regularly Interspaced Short Palindromic Repeats/CRISPR associated protein (CRISPR/Cas) gene editing speed up entire gene function analysis [18]. Most genetic transformations in plants are mediated by *Rhizobium radiobacter* (formerly known as *Agrobacterium tumefaciens*), a phytopathogenic bacterium capable of integrating its transfer-DNA (T-DNA) into the plant genome to express tumor-inducing and opine synthesis genes, which support its survival [19]. These genes on the T-DNA can be substituted by any desired gene [20]. Typically, *R. radiobacter*-mediated stable transformation protocols are based on in vitro regeneration protocols [18]. Consequently, developing molecular genetic tools for a novel species starts with establishing a tissue culture protocol. For a transformation protocol, a de novo regeneration of plants via organogenesis or somatic embryogenesis is essential to achieve stable transformants while reducing chimeric regenerants [21]. De novo organogenesis refers to the novel differentiation of meristematic tissue from somatic cells, either directly or via a callus phase [22]. However, such a regeneration protocol has not yet been established for *N. amplexicaulis*.

In the subfamily Mimosaceae, only three micropropagation protocols were published for *Acacia ceasia*, *Mimosa pudica* and *N. amplexicaulis* in total [23–25]. For *Mimosa pudica*, also successful genetic transformation was described [26]. So far, only one de novo organogenesis protocol has been published for the Mimosaceae species *Neptunia oleracea* [27]. Yet, the micropropagation protocol from O´Donohue et al. [25] shows that in vitro establishing of *N. amplexicaulis* is possible. In their study, shoot tips and axes were cut off in vitro germinated seedlings and placed on Murashige and Skoog (MS) medium [28] supplemented with 6-benzylaminopurin (BAP). Multiplication rates reached a maximum of two shoots per node on medium containing 2 mg/L BAP and 0.2 mg/L naphthalene acetic acid (NAA), accompanied by a reduction in shoot length from 4.5 cm to 1.9 cm compared to explants grown on MS medium without PGRs. Transferring the shoots to medium with or without 0.02 mg/L NAA induced roots at rates of 15–30% after 4 weeks. 95% of rooted shoots survived acclimatization. Thus, MS medium is a valid start for a culture and provides an orientation for developing media for de novo regeneration and Se hyperaccumulation in *N. amplexicaulis*.

Thus far, only a few pioneering studies exist in approaching functional genetics in hyperaccumulator species yet, for instance successful transformation was reported for the nickel (Ni) hyperaccumulator *Sedum plumbizincicola* and the cadmium (Cd) hyperaccumulator *Arabidopsis halleri* [29, 30]. For the zinc (Zn)/Cd/Ni hyperaccumulator *Noccaea caerulescens* a mutant library was created and a transcriptome of the Se hyperaccumulator *S. pinnata* was sequenced [14, 31]. In conclusion, the field of hyperaccumulation urgently awaits genetic approaches. Engineering molecular tools for *N. amplexicaulis* will speed up research into the genetic basis of hyperaccumulation, elucidating theories about the evolution of Se hyperaccumulation and developing strategies for Se biofortification programs. The aims of this study were (i) to develop a de novo regeneration protocol for *N. amplexicaulis* and (ii) to establish conditions to test for Se accumulation under in vitro conditions. Thereby, the establishment of *N. amplexicaulis* as a model species for studying Se hyperaccumulation should be facilitated.

## Material and methods

### Origin and storing of *N. amplexicaulis* and *N. heliophila* seeds

The *N. amplexicaulis* seeds used, were an offspring from a population grown at the University of Queensland, St Lucia campus, on 28 mg Se kg^−1^ as selenate dosed soils. *N. heliophila* seeds were from the same source but cultivated without the Se dosing. The population on the St Lucia campus was established from seeds collected in Richmond, Queensland in 2018 [11]. Seeds were stored at room temperature excluded from light. The growth conditions for the *Neptunia* species are explained for every conducted experiment, separately.

### Composition and preparation of tissue culture media

Most media were based on the original MS medium [28] and their detailed composition is given in Table 1. For preparing the media, Murashige & Skoog Medium including vitamins (Duchefa, Netherlands) powder or MS Medium Modification No. 3A: ½ concentration of NH_4_NO_3_ and KNO_3_, were used. After adding all autoclavable components to the medium, the pH was adjusted before adding Phyto Agar (Duchefa, Netherlands). All media were autoclaved at 121 ± 1 °C for 20 min. Non-autoclavable components where filter-sterilized with a 0.22 µM PVDF membrane filter (Whatman^®^; UK) and added after autoclaving when the temperature of the medium was below 60 °C. Media names follow a specific pattern: the first part of the name indicates the purpose of the medium (e.g., SIM for Shoot Induction Medium) and the basal composition denoted by a number. The second part consists of an abbreviation of the growth regulator used and its concentration in mg/L (Table 1).

**Table 1:** Composition of media used for germination (Germ), shoot induction (SIM) and shoot regeneration and elongation (SRM). The pH was adjusted with KOH as the final step before adding Phyto agar and autoclaving

Compound	Germ-1	SIM9-BAP2	SIM9-BAP0.2	SIM9-TDZ1 + Se	SIM9-TDZ0.1 + Se	SIM9-TDZ1 + Glu	SIM9-TDZ0.1 + Glu	SIM10-TDZ1	SIM10-TDZ0.1	SRM1-BAP1	SRM1-BAP0.5 + Se	SRM2-GA_3_1	In vitro Se standard medium
MS Micro and Macro elements incl. Vitamins	2.2 g/L	–	–	–	–	–	–	–	–	–	–	–	4.4 g/L
MS mod. No.3A Micro and Macro elements, ½ conc. NH_4_NO_3_ and KNO_3_	–	4.4 g/L	4.4 g/L	4.4 g/L	4.4 g/L	4.4 g/L	4.4 g/L	2.5 g/L	2.5 g/L	4.4 g/L	4.4 g/L	4.4 g/L	–
B5 vitamins	–	112 mg/L	112 mg/L	112 mg/L	112 mg/L	112 mg/L	112 mg/L	112 mg/L	112 mg/L	112 mg/L	112 mg/L	112 mg/L	–
Sucrose	–	20 g/L	20 g/L	20 g/L	20 g/L	20 g/L	20 g/L	20 g/L	20 g/L	20 g/L	20 g/L	20 g/L	–
MES monohydrate	0.4 g/L	0.4 g/L	0.4 g/L	0.4 g/L	0.4 g/L	0.4 g/L	0.4 g/L	0.4 g/L	0.4 g/L	0.4 g/L	0.4 g/L	0.4 g/L	0.4 g/L
Phyto agar	7.5 g/L	7.5 g/L	7.5 g/L	7.5 g/L	7.5 g/L	7.5 g/L	7.5 g/L	7.5 g/L	7.5 g/L	7.5 g/L	7.5 g/L	7.5 g/L	7.5 g/L
BAP	–	8.9 µM	0.89 µM	–	–	–	–	–	–	4.5 µM	2.3 µM	–	–
TDZ	–	–	–	4.5 µM	0.45 µM	4.5 µM	4.5 µM	4.5 µM	0.45 µM	–	–	0.45 µM	–
NAA	–	1.1 µM	-	–	–	–	–	–	–	–	0.5 µM	–	–
GA_3_	–	–	–	–	–	–	–	–	–	–	–	3 µM	–
NaSeO_3_	–	–	–	10 µM	10 µM	–	–	–	–	–	10 µM	–	–
Na_2_SeO_4_	–	–	–	10 µM	10 µM	–	–	–	–	–	10 µM	–	0–90 µM
STS	–	–	–	50 µM	–	–	–	–	–	–	–	–	–
L-glutamine	–	–	–	–	–	1 mM	2.5 mM	–	–	–	–	–	–
pH	5.8	6.5	6.5	6.5	6.5	6.5	6.5	6.5	6.5	6.5	6.5	6.5	5.8

### Seed surface disinfection and scarification of *N. amplexicaulis*

First, one cut was made on one lateral side of the seed with a scalpel through the seedcoat. Further steps were performed under sterile conditions in a flow cabinet. A maximum of 50 seeds were transferred to a sterile 50 mL reaction tube and rinsed once with 70% ethanol (EtOH) for 30 s, gently shacken and rinsed once with sterile tap water. Thereafter, the seeds were submerged in 2.4% sodium hypochlorite and incubated for 12 min. After incubation, the hypochlorite was discarded, and the seeds were washed three times with sterile tap water. For the fourth wash, sterile tap water was added, and the seeds were left to soak therein in the dark at room temperature for at least 8 h. Swollen seeds were placed on Germ-1 unless otherwise specified.

### Seed surface disinfection, scarification and stratification of *M. truncatula*

Seeds were removed from the seed pods by striking it once with a hammer. The broken capsules were scratched on a metal sieve and the individual *M. truncatula* seeds were collected. The desired number of seeds were filled into a 12 mL reaction tube. 2–4 mL of 96% sulphuric acid was added to the seeds and the seeds were incubated for 12 min. Then, the seeds were rinsed with sterile tap water at least three times. Next, seeds incubated in 2.4% sodium hypochlorite for 1 min and rinsed again three times with sterile water. After rinsing, the seeds were kept in sterile water to soak for 4 h. Swollen seeds were placed on Germ-1 and stratified at least for two days at 4 °C in the dark.

### Growth conditions and explant preparation

**Fig. 1:**
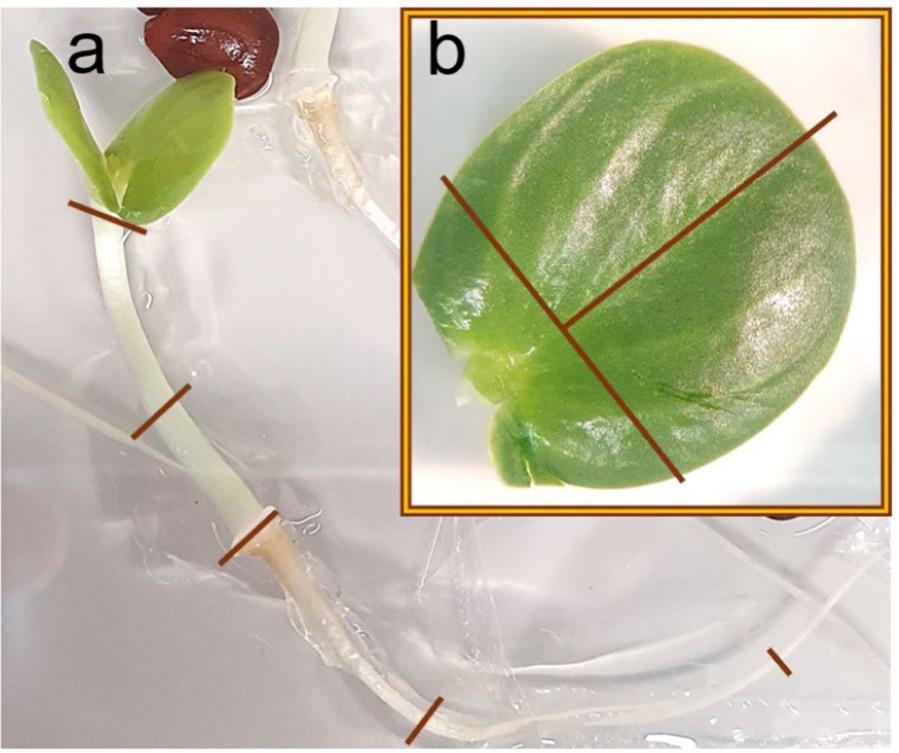
Explant preparation from seedlings. **a** Image of a 5-day old *N. amplexicaulis* seedling on ½ MS medium showing where the seedlings hypocotyl, radicle and **b** cotyledons were cut before placing on SIM

Seeds of *N. amplexicaulis* were germinated on Germ-1 (Table 1) over 5 days: initially for 2 d in darkness, followed by 3 d at 27.5/26 °C under a 16/8 h day/night cycle with an intensity of 100 $\frac{\mu mol}{{m}^{2}*s}$. This experiment was conducted in a growth chamber (Memmert ICH260L, Germany). Each cotyledon was cut into three parts: the first cut was transversal to the veins, resulting in an explant containing the petiole, and the other part of the cotyledon was cut longitudinally through the middle vein (Fig. 1). The resulting six cotyledonary explants of one seedling, were placed in one Petri dish. Six to seven root and hypocotyl explants pooled from multiple seedlings were used per Petri dish.

First, explants were incubated at 27.5/26 °C (day/night) and a relative humidity of 60% for 3 ½ weeks in the dark, then they were exposed to light with an intensity of 100 $\frac{\mu mol}{{m}^{2}*s}$ in a 16/8 h day/night cycle.

### Experimental set-up for regeneration experiments

Three treatments were compared in regeneration experiment 1, each defined by a succession of two media (Table 2). Explants were incubated on media SIM9-BAP2, SIM9-TDZ1 or SIM9-TDZ1 + Se (Table 1) for two weeks and then transferred to media with cytokinin concentrations reduced to 1/10 respectively (Table 2). Thereafter, this medium was refreshed every two weeks. Callus formation and explant browning of the explants were assessed by visual rating (Supplemental Figure S1) and with an image analysis (see below) after 4 and 8 weeks. In regeneration experiment 2, the initial dark incubation period was reduced to 3 weeks. The BAP-variant was not included again, instead two new variants were included, SIM10-TDZ1/0.1 and SIM9-TDZ1/0.1 + Glu, to analyze the effect of reduced nutrient concentrations and glutamine on regeneration frequency (Table 2). In this experiment, numbers of hypocotyl and root explants were increased, because no shoot regeneration had been recorded on cotyledon explants in regeneration experiment 1.

**Table 2:** Replicate numbers and medium succession for the variants of the regeneration experiments. The number of Petri dishes and explants for each explant type are listed for each variant

		Number of Petri dishes (explants)				
Variant	Replicates (Petri dishes) in total	Cotyledons	Hypocotyls	Roots	Initial medium (2 weeks)	Successive medium (3 × 2 weeks)
Regeneration experiment 1						
SIM9-BAP2/0.2	8	6 (38)	1 (7)	1 (6)	SIM9-BAP2	SIM9-BAP0.2
SIM9-TDZ1/0.1	8	6 (38)	1 (7)	1 (8)	SIM9-TDZ1	SIM9-TDZ0.1
SIM9-TDZ1/0.1 + Se	8	6 (36)	1 (6)	1 (7)	SIM9-TDZ1 + Se	SIM9-TDZ0.1 + Se
Regeneration experiment 2						
SIM10-TDZ1/0.1	9	6 (36)	3 (9)	3 (10)	SIM10-TDZ1	SIM10-TDZ0.1
SIM9-TDZ1/0.1	7	5 (30)	2 (6)	2 (10)	SIM9-TDZ1	SIM9-TDZ0.1
SIM9-TDZ1/0.1 + Se	8	6 (36)	2 (7)	2 (7)	SIM9-TDZ1 + Se	SIM9-TDZ0.1 + Se
SIM9-TDZ1/0.1 + Glu	8	5 (29)	3 (10)	3 (11)	SIM9-TDZ1 + Glu	SIM9-TDZ0.1 + Glu

### Further cultivation of the de novo shoot buds

De novo shoot buds detected after 6 weeks of cultivation, were separated from their explants and further cultivated on SIM9-TDZ0.1 or SIM9-TDZ0.1 + Se for two weeks. Brown callus parts were cut off from the explants. When clear shoot outgrowth was detected after 8 weeks, explants were transferred to SRM1-BAP0.5 (Table 1) to promote shoot outgrow and elongation. After 9 weeks, the explants were moved to SRM1-BAP1 (Table 1). Weekly photographs were taken of the small shoots, and their growth and browning were assessed based on these images and direct observation. Finally, after 11 weeks, they were transferred to SRM2-GA_3_1 (Table 1).

### In vitro Se tolerance and accumulation test

For this experiment, MS medium with 15 times reduced S content was needed (Table 1). Thus, instead of 1.5 mM MgSO_4_·7H_2_O, only 0.1 mM MgSO_4_·7H_2_O was used in the media with low S concentration. Therefore, the MS medium was prepared from single stock solutions. The treatments were MS medium and 1/15xS_MS medium, each supplemented with 0 μM (control), 30 μM, and 90 μM sodium selenate (Na_2_SeO_4_). Medium with reduced S was additionally supplied with 1 mM MgCl_2_·6H_2_O for balancing the magnesium (Mg) level. The 12 × 12 cm square Petri dishes were placed at an angle of 15° before the medium was poured until it reached the edge of the Petri dish. Seedlings of *M. truncatula*, *N. amplexicaulis*, and *N. heliophila* were germinated for 7 d as described. Two seedlings were placed on each dish with the transition zone of the root and hypocotyl positioned in the middle of the plate, with the flat agar side on top and the medium-rich side at the bottom. For every species 3 Petri dishes were prepared per medium. Petri dishes were closed with one layer of parafilm and stacked at an upright 90° angle in a cassette, so that the upper quarter of the plate was in the light and the rest of the Petri dish was shaded by the cassette and box. Explants were grown on these plates at 24 °C under 100 μ*mol*/ *m*^2^ ∗ *s* with a 16/8 h day/night cycle for 4 weeks. Photos of the Petri dishes were taken at 0, 7 and 28 d (at harvest).

### Grinding of plants and metallomics measurement.

Plants grown for 4 weeks on Petri dishes were completely removed from the agar and carefully cleaned. The plants were then drained on kitchen paper, divided into root and shoot segments, and placed in 1.5 mL reaction tubes containing three 2 mm and two 3 mm ceramic beads. The fresh mass of the root and shoot segments was recorded. For drying, the tube lids were left open, and the plants were incubated in an oven at 60 °C for 3 days. The tubes were then weighed again to determine their dry mass. Due to the low mass of the root explants, all root samples from one treatment were pooled. After drying, all samples were cooled at − 80 °C. The samples were ground using a mixer mill (Retsch, Germany) for 2 min at 25 Hz. In preparation for analysis using the metal analyzer Z-Spec JP500 (USA), the tip of the measurement tube was covered with a 6 μm polypropylene foil. The powder was placed on this foil and covered with a second layer of foil. Each sample was measured in "leaf" mode for 60 s.

### Image and statistical analysis

Image analysis of the Petri dishes of tissue culture explants for scoring the callus formation and tissue browning was performed with ImageJ [32]. The color threshold was used to select the complete tissue area of all explants on one Petri dish. As the next step, only the brown part of all explants was selected via color threshold and the callus area was encircled manually. The relative callus and browned area of an explant were calculated by dividing the browned or callus area by the total explant area. Statistical analyses were performed in R 4.2.2 using RStudio [33]. The image data was analyzed in a two factorial ANOVA and afterwards in a post hoc Tukey test, if the ANOVA showed significant (*p* < 0.05) effects. Prior to the ANOVA, image data was tested for normal deviation with a Shapiro–Wilk normality test and for variance homogeneity with a Barlett test to legitimate its conduction. The packages “ggplot2” and “dpyr” were applied for visualizing plots in R [34, 35].

## Results

### The medium of de novo shoot regeneration

A series of experiments was conducted to establish the early stages of a de novo regeneration protocol for *N. amplexicaulis*. Therein, the basal medium composition as well as plant growth regulator combinations and concentrations were varied along with physical growth conditions (data not shown). As an outcome, the medium SIM9 was developed consisting of an increased nutrient concentration compared to MS (except for the nitrogen sources) (Table 1) along with B5 vitamins [36] and a pH value of 6.5. This medium was further optimized in the two regeneration experiments presented in this study.

### Callus formation and browning of explants (regeneration experiment 1)

**Fig. 2:**
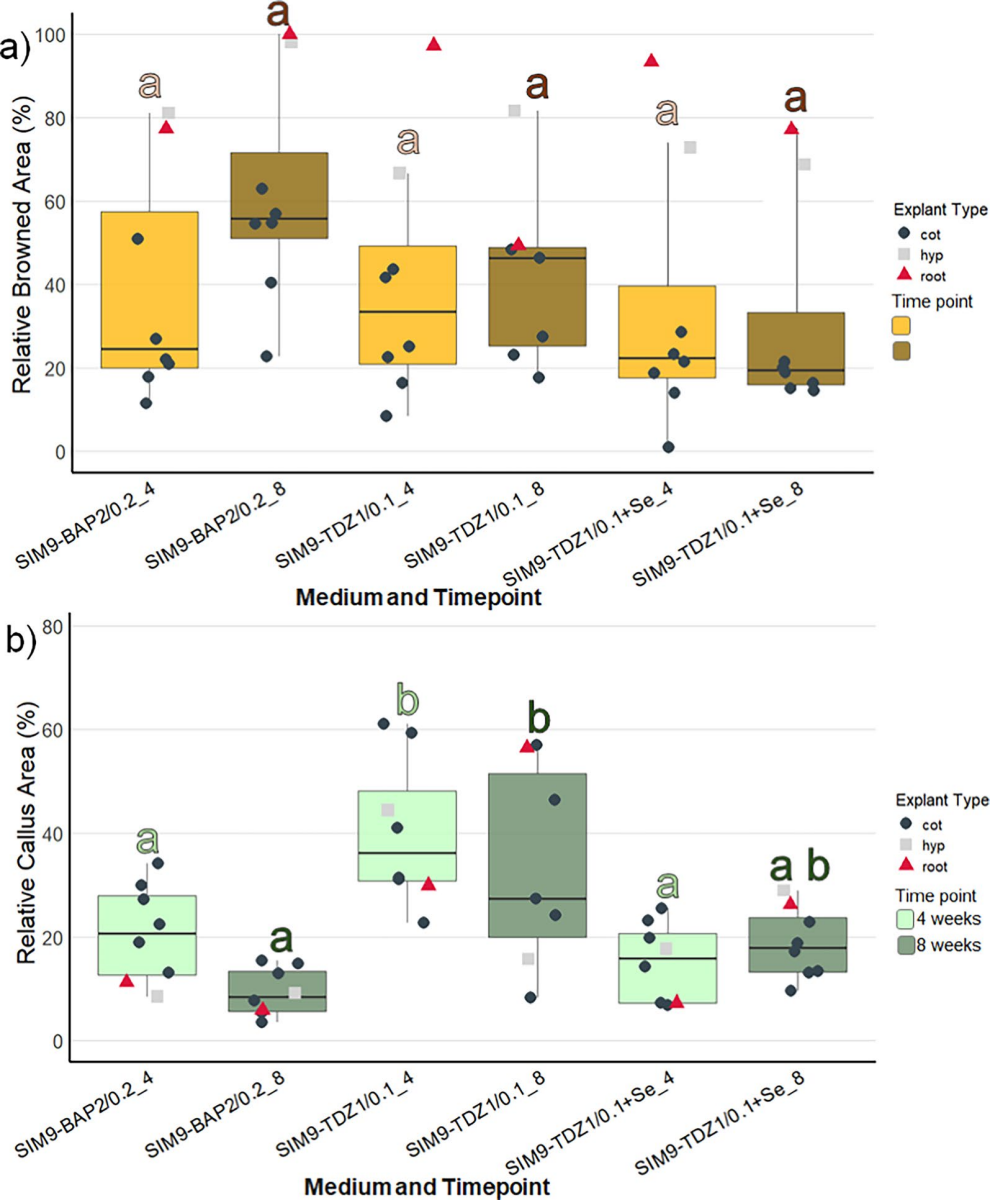
Browning and callus formation of *N amplexicaulis* seedling explants of regeneration experiment 1 after 4 and 8 weeks of culture. Medium variants are displayed in Table 2. The data points summarize the explants values from one Petri dish. The shape and color of the data points indicate different explant types: cot = cotyledon, hyp = hypocotyl and root = root explants. The box plots display the median and cover the interquartile range. Letters above the bars indicate significance levels (*p* < 0.05) based on a Tukey test; n ≤ 9. The test was conducted separately for data collected at 4 and 8 weeks. **a** The relative browned area of the explants. **b** The relative callus area of the explant

Three media were compared differing in the cytokinin type and concentration (4.5 µM TDZ and 8.9 µM BAP) as well as the addition of Se (Table 2). As the explants were kept in the dark for 3 weeks, the initial callus produced was transparent or white. Within the first few days after transfer to light, the callus turned green, so that at the 4-week screening, all the callus cultures appeared green. In general, the image data (Fig. 2) aligned well with the visual categorization results (Figure S2) and allowed the statistical analysis of metric data. Therefore, the following description focuses on the image data: Explant browning after 4 weeks was generally low and not significantly different between the media (Fig. 2a, Table S1). During the next 4 weeks, browning of explants increased only slightly on SIM9-TDZ1, whereas it strongly increased on SIM9-BAP2. This difference was not statistically significant according to the post-hoc test applied (Fig. 2b, Table 3), although the ANOVA for browning after 8 weeks revealed a significant effect of the medium (Table S1). Strong callus formation was observed medium SIM9-TDZ1 already after 4 weeks resulting in a significantly larger relative callus area than observed for explants on SIM9-BAP2 (Fig. 2b). Callus growth on both TDZ-containing media continued, while explants on the BAP containing medium remained unchanged compared to the evaluation after 4 weeks (Fig. 2).

**Table 3:** Number of *N. amplexicaulis* root and hypocotyl explants that showed the initiation of de novo shoot bud regeneration in regeneration experiments 1 and 2

Variant	Number of hypocotyl explants	Number of differentiating hypocotyl explants	Number of root explants	Number of differentiating root explants
Regeneration experiment 1				
SIM9-BAP2/0.2	7	0	6	0
SIM9-TDZ1/0.1	7	1	8	0
SIM9-TDZ1/0.1 + Se	6	1	7	1
Regeneration experiment 2				
SIM10-TDZ1/0.1	9	1	10	0
SIM9-TDZ1/0.1	6	1	10	0
SIM9-TDZ1/0.1 + Glu	10	0	11	0
SIM9-TDZ1/0.1 + Se	7	0	7	0

When comparing the different explant types (represented by the shape and color of data points in Fig. 2), callus formation within the same medium was similar after 4 weeks of culture. However, the callus formed on root explants on SIM9-TDZ1, almost doubled in relative callus area between 4 and 8 weeks. On the other hand, root as well as hypocotyl explants were affected strongly by browning on all media already after 4 weeks. New browning occurred only on the older callus parts of the explants, while the fresh callus remained unaffected and green.

### First evidence for de novo regeneration of shoot buds (regeneration experiment 1)

**Fig. 3:**
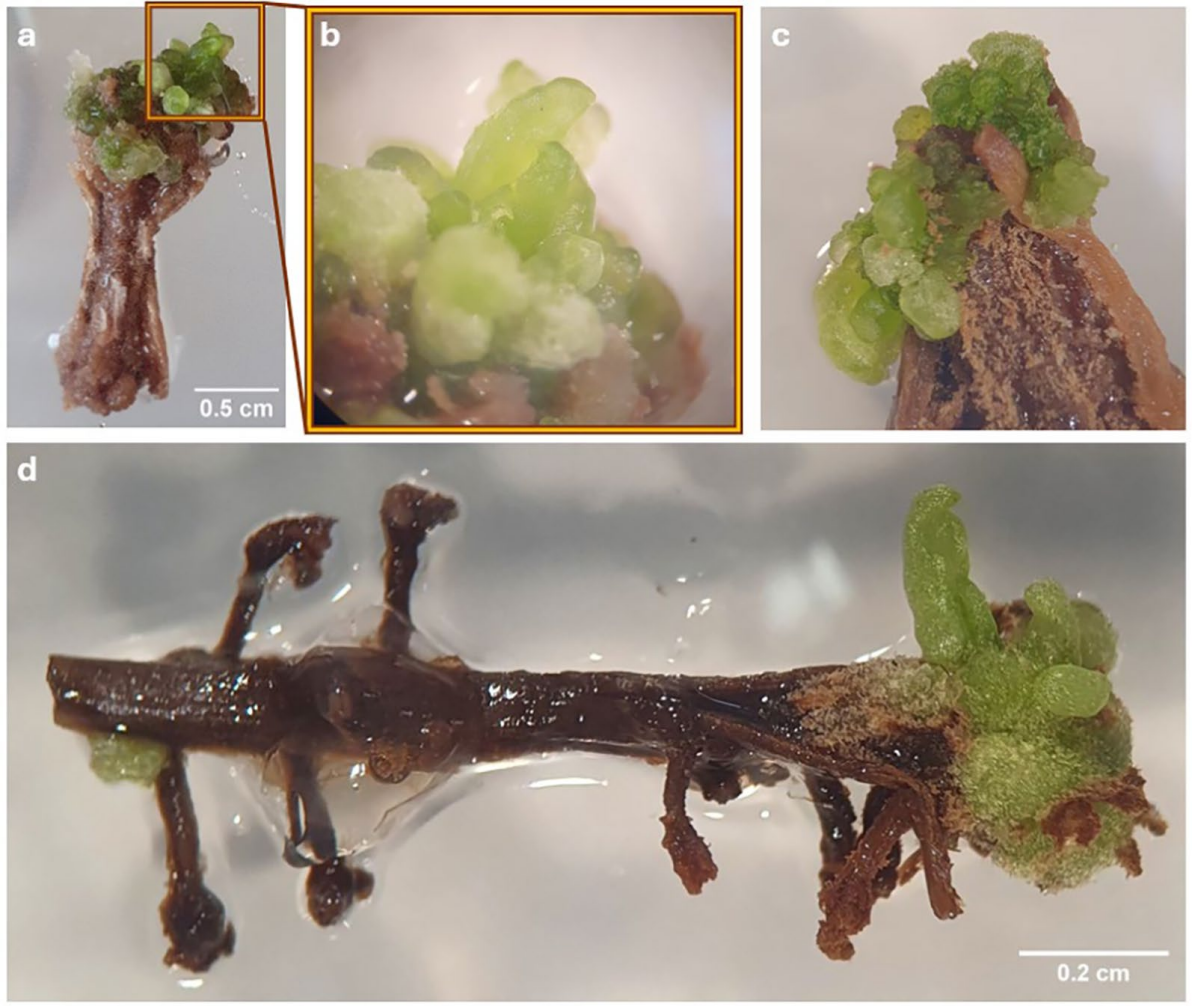
Initial stadium of de novo shoot differentiation on *N. amplexicaulis* explants. **a** Hypocotyl explant after 6 weeks on SIM9-TDZ1/0.1. **b** Close-up of **a** showing the differentiating shoot bud. **c** Hypocotyl explant after 6 weeks on SIM9-TDZ1 + Se with differentiating structures within callus. **d** Root explant after 6 weeks on SIM9-TDZ1 + Se with the root tip forming leaves

**Fig. 4:**
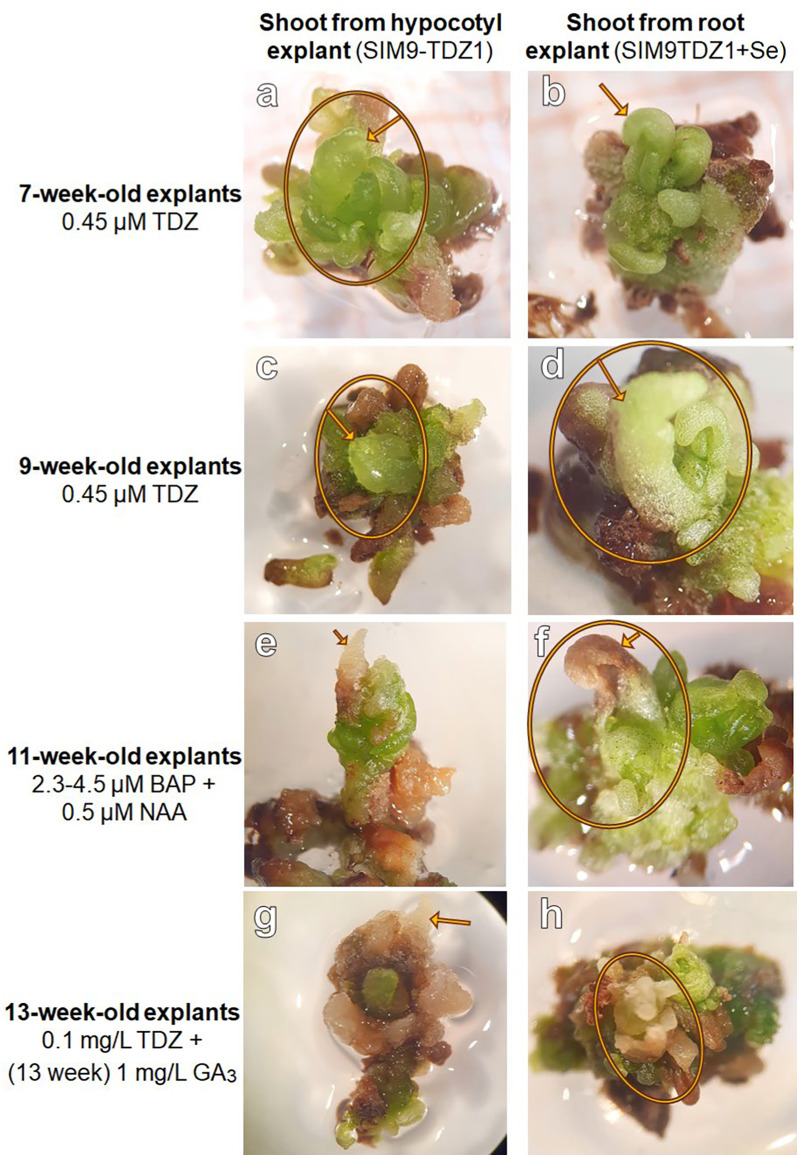
De novo shoot formation in *N. amplexicaulis* explants and further development on media containing different PGR combinations. This chronology showed the further development during which the PGRs were modified as described on the left. The arrow points always to the same shoot bud tip

During a screening of the explants, 6 weeks after placing the explants on the media, small differentiating structures with a smooth surface were detected on a single hypocotyl explant on SIM9-TDZ1 and on a root and a hypocotyl explant on SIM9-TDZ1 + Se (Fig. 3a–d). The structures resembled multiple small shoots or pinnate leaflets of *N. amplexicaulis,* but their morphology was affected by hyperhydricity symptoms (Fig. 3b) hindering a more precise description. After 8 weeks of culture, the number of differentiating explants had not increased. The regeneration rate for roots, calculated based on the number of root explants placed on media where regeneration was observed, was 1 out of 15 (6.6%), while the regeneration rate for hypocotyls was 2 out of 13 (15.4%) (Table 3). The low total number of root and hypocotyl explants used in this experiment which focused on cotyledon explants did not allow a statistical analysis. The hypocotyl explant on SIM9 TDZ1_0.1 + Se (Fig. 3c) did not differentiate further. Separating the rudimental differentiating shoot buds from the original explant and keeping them on the same media for two more weeks led to an increase in the size of the explants without the formation of additional shoot buds (Fig. 4). For the root explant on SIM9 TDZ1_0.1 + Se, growth ceased after transferring the explants to a medium containing 2.3 µM BAP, followed by 4.5 µM BAP and 0.5 µM NAA. Browning occurred, starting from both the top and the bottom of the explant. In general, browning occurred on the explant surfaces in contact with the medium already after 2 days.

For the remaining shoot forming hypocotyl explant, growth was also inhibited; however, the shoot bud elongated slightly. When the explants were placed back on SIM9-TDZ0.1, growth resumed slightly, and new shoot buds emerged from the explants. Two weeks after transfer to medium containing 3 µM gibberellic acid (GA_3_), no elongation or further differentiation was observed. Instead, the differentiated structures became more brownish and callus-like, while new green tissue appeared around or adjacent to the old tissue without forming additional structures (Fig. 4). So far, the regeneration of complete shoots has not been achieved.

### Reproduction of initial shoot regeneration in *N. amplexicaulis* (regeneration experiment 2)

**Fig. 5:**
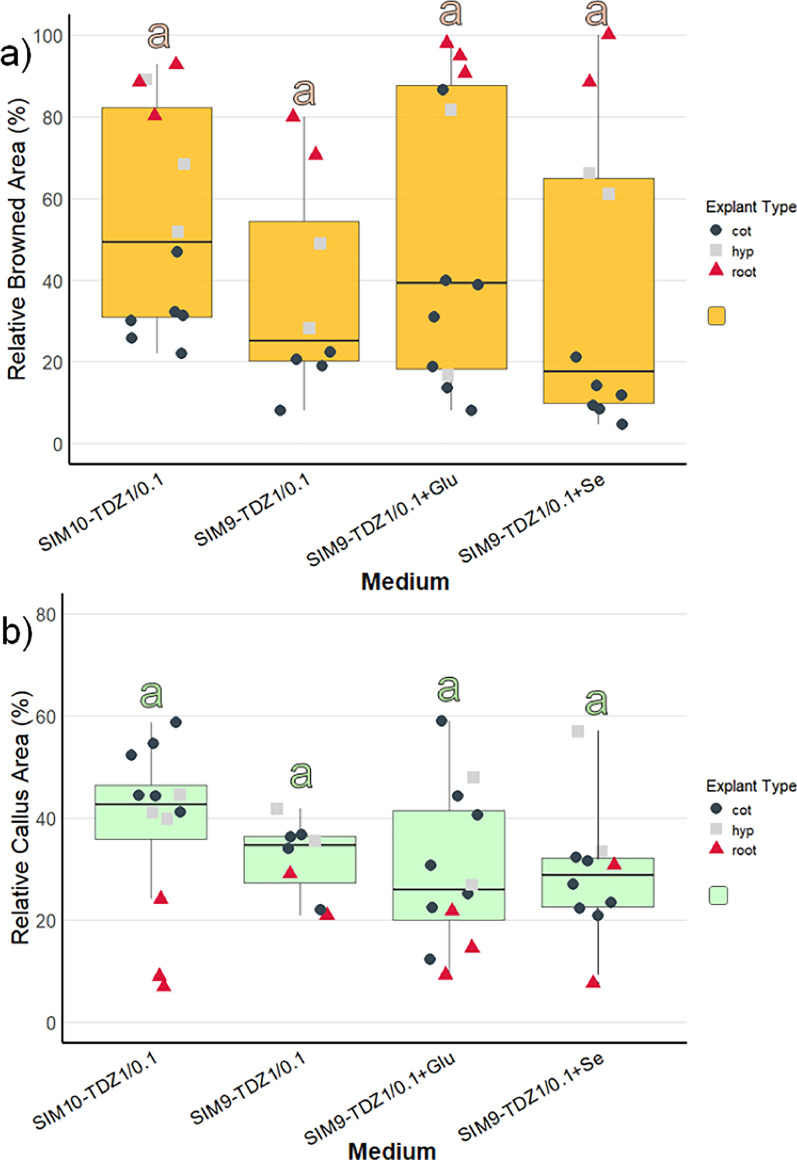
Browning and callus formation of *N amplexicaulis* seedling explants of regeneration experiment 2 after 4 weeks of culture. Medium variants are displayed in Table 2. The data points summarize the explants values from one Petri dish. The shape and color of the data points indicate different explant types: cot = cotyledon, hyp = hypocotyl and root = root explants. The box plots display the median and cover the interquartile range. Letters above the bars indicate significance levels (*p* < 0.05) based on a Tukey test. **a** The relative browned area of the explants. **b** The relative callus area of the explants

Since the nascent shoot formation in regeneration experiment 1 was only observed on hypocotyl and root explants, the number of these explants was increased in regeneration experiment 2. Due to time constraints, the experiment had to be finalized after 6 weeks culture. Across the four media, callus formation was most pronounced on SIM10-TDZ1, a MS medium with ½ concentrated NH_4_NO_3_ and KNO_3_, although differences in callus formation between media were not statistically significant (Fig. 5, Suppl. Table S2). The weakest callus formation, along with the least browning, was observed on SIM9-TDZ1 + Se, consistent with results from regeneration experiment 1. The supplementation of glutamine in SIM9-TDZ1 + Glu neither improved nor reduced callus formation or browning, with the observed development averaging between that of the other media.

However, within individual media treatments, callus induction was stronger in cotyledons and hypocotyls compared to roots (Fig. 5a). For tissue browning, the medium composition influenced browning significantly (Supp. Table S2) and was strongest on SIM10-TDZ1, but post-hoc tests did not reveal any significant pairwise differences (Fig. 5b). The difference in browning between explant types was pronounced (Suppl. Table S3). Cotyledons exhibited the lowest browning, with less than 40% of the tissue affected (Fig. 5a, Suppl. Table S3). Hypocotyls showed a wider range, with browned areas varying between 20 and 80%. In contrast, root explants were mostly browned, with over 80% of the tissue affected, except the root explants on SIM9-TDZ1.

**Fig. 6:**
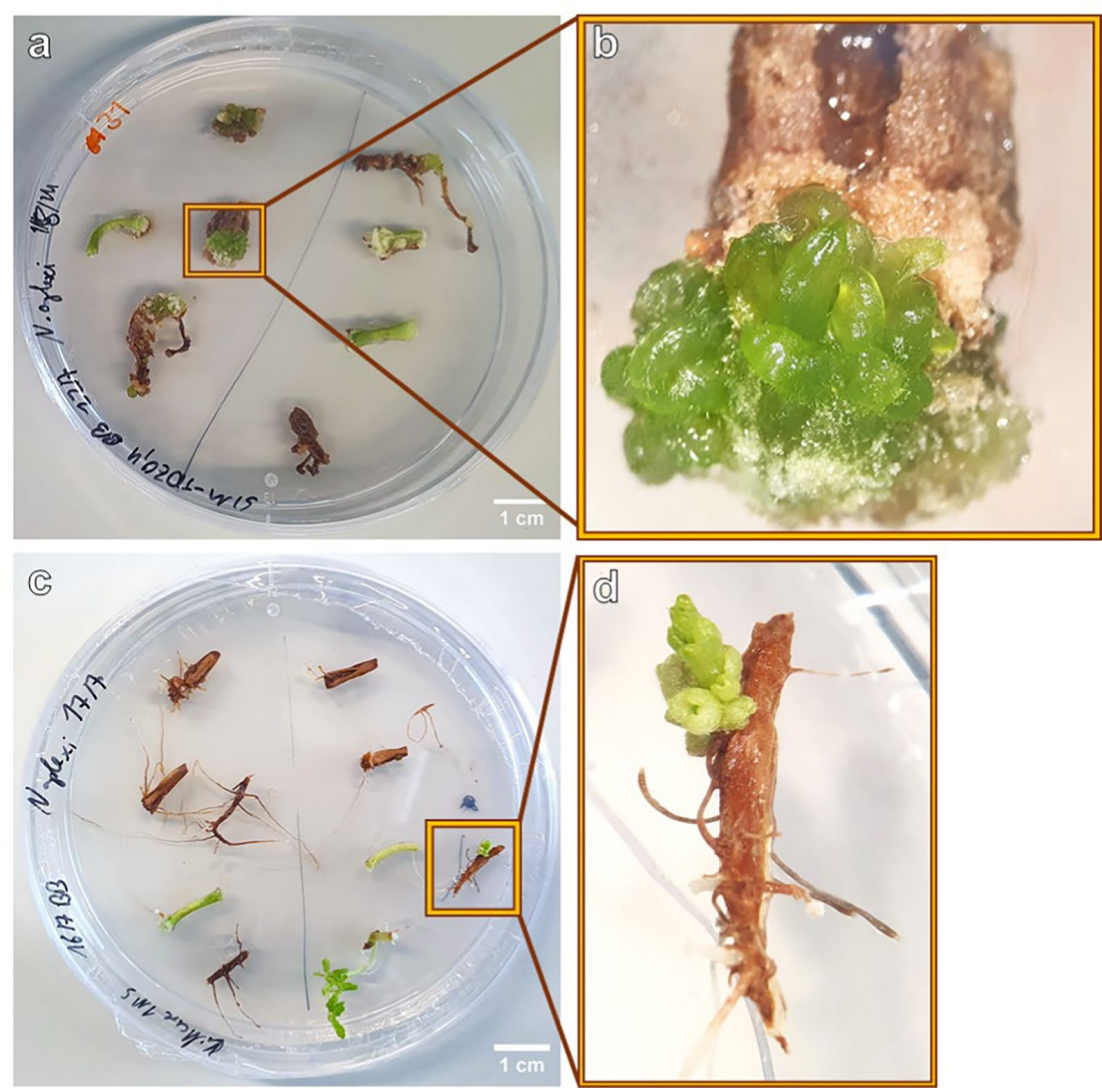
**a** Image of a Petri dish with 6-week-old *N. amplexicaulis* hypocotyl and root explants of the regeneration experiment 2, variant SIM9-TDZ1/0.1. **b** Close-up of **a** highlighting the hypocotyl explant with shoot but development. **c** Root and hypocotyl explants of *N. amplexicaulis* after 4 weeks on MS medium without PGRs, grown in light at 27.5 °C. **d** Close-up of **c** with the individual root explants regenerating a shoot bud

Initial shoot bud differentiation was observed after 6 weeks on two hypocotyl explants (Fig. 6a, b). On one hypocotyl explant each cultured on medium SIM9-TDZ1 and on SIM10-TDZ1, multiple shoot buds developed from the callus (Table 3). Notably, the explants displayed a deeper green coloration, which was clearly distinguishable from the surrounding non-differentiating callus and had been characteristic also for the shoot buds observed in regeneration experiment 1. However, not all explants with callus of this color differentiated shoot buds. In summary, in this experiment the differentiation of shoots was reproduced on media containing TDZ.

### Spontaneous shoot regeneration on *N. amplexicaulis* roots

A discovery was made outside of the two regeneration experiments shown here, on a control Petri dish with MS medium without any PGRs. Six weeks after placing the explants on the medium, one root explant regenerated a shoot (Fig. 6c, d). In particular, the single root explant differentiated a new matte green bud structure, similar to the differentiation observed in the two regeneration experiments. Furthermore, this bud clearly showed leaves, and the root explant also initiated lateral roots at the base (Fig. 6d).

### In vitro test system for Se accumulation

**Fig. 7:**
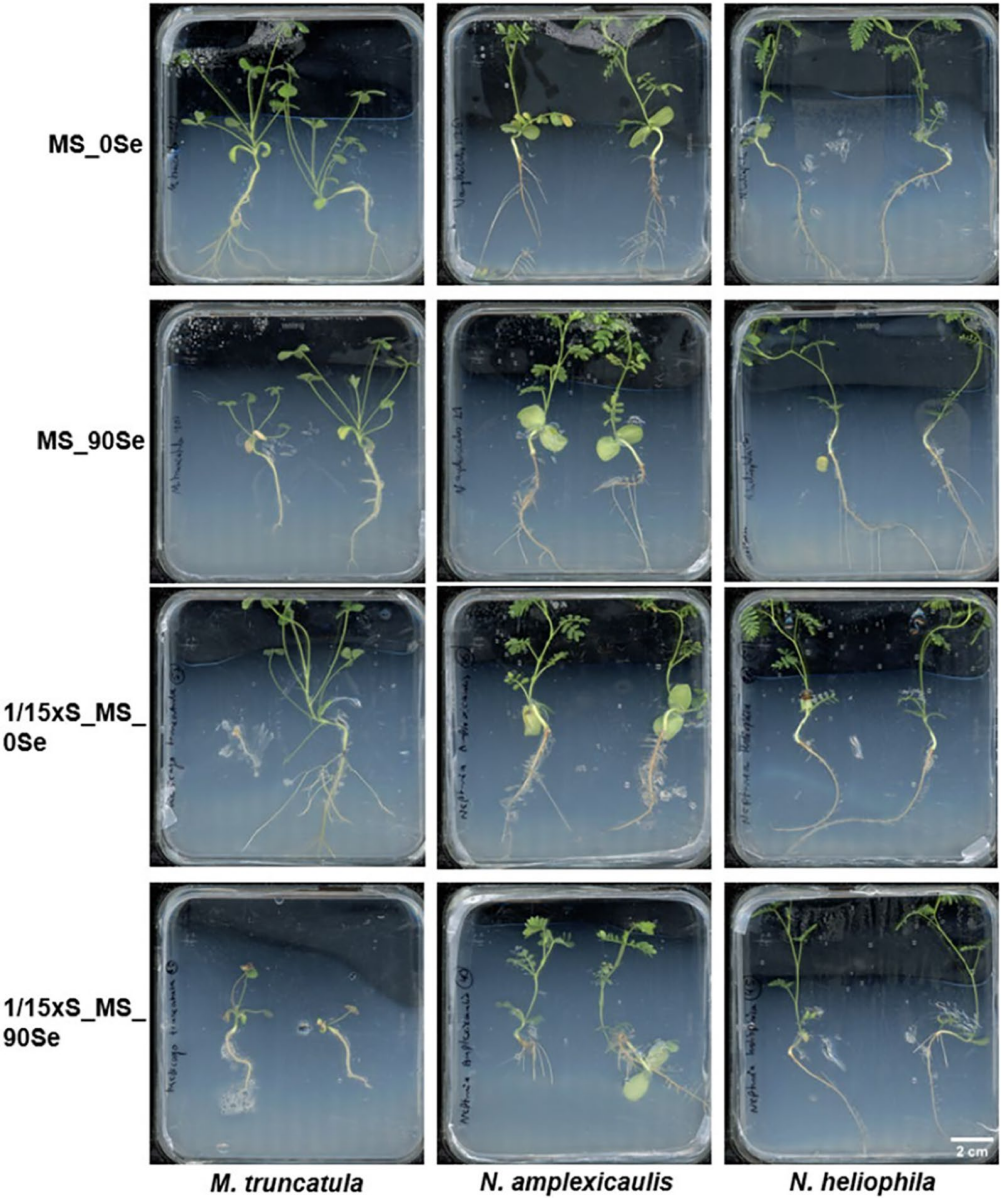
Seedlings of *M. truncatula, N. amplexicaulis* and *N-heliophila* after 4 weeks of culture on media differing in S and Se concentrations. Scans of representative Petri dishes are shown with all seedlings being placed with the root neck in the middle of the dishes

**Fig. 8:**
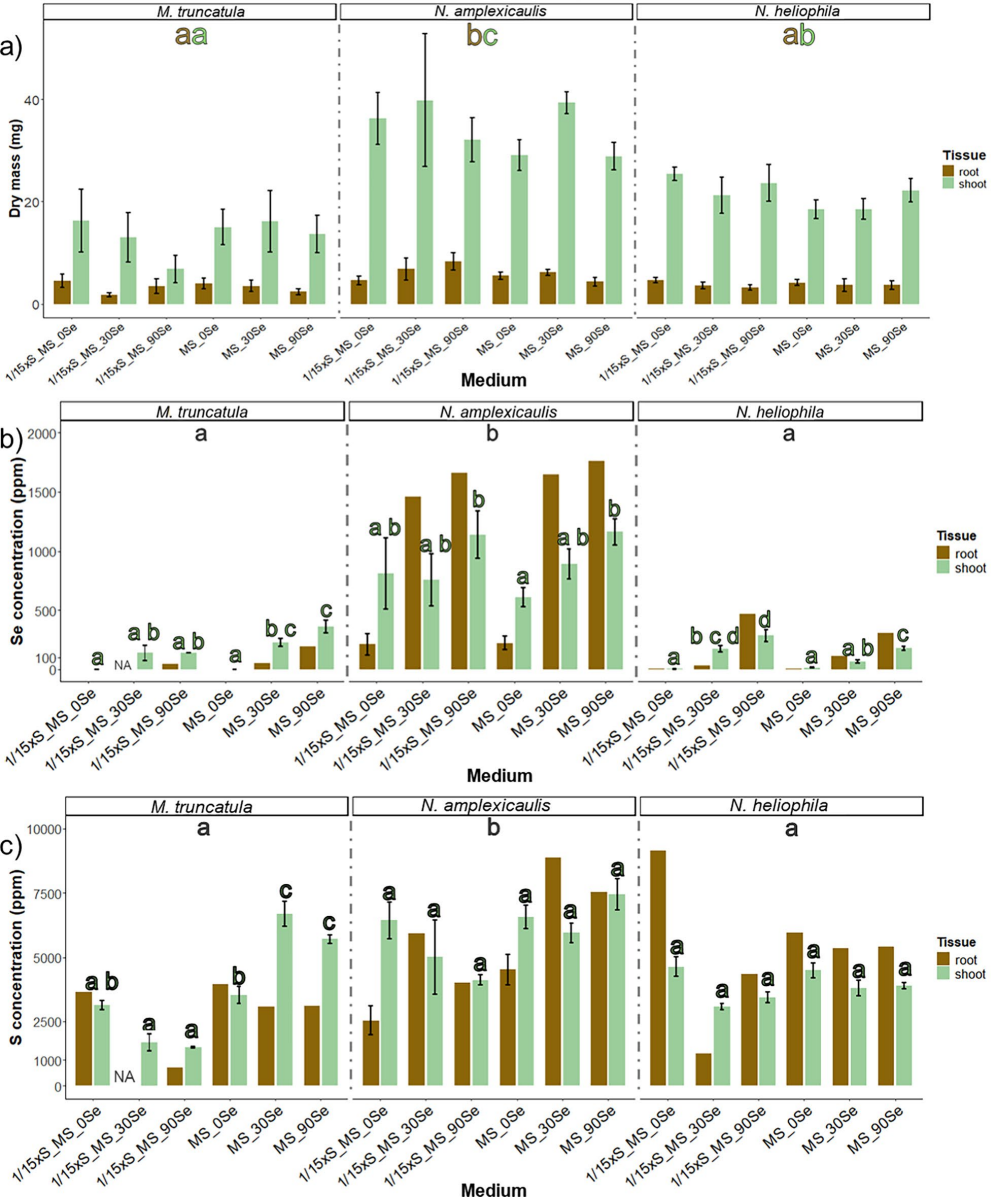
Effect of different S and Se concentrations in culture media on dry mass (**a**) and Se (**b**) and S (**c**) concentrations of shoots and roots of *M. truncatula, N. amplexicaulis* and *N-heliophila* seedlings after 4 weeks of culture. The error bars demonstrate SEs in a) and SD in **b** and **c**. The letters indicate statistically significant differences between the species (*p* < 0.05) based on a pairwise Tukey test, shown separately for roots (brown letters) and shoots (green letters); n = 6. Letters indicating differences within a species between media are shown directly on top of the bar for shoot data only. Root samples were pooled resulting in only one value per medium

The objective of this experiment was to establish an in vitro test system which enables the investigation of Se uptake and accumulation in seedlings. Therefore, seedlings of three Fabaceae species were grown on media with different Se concentrations and with full or reduced S concentration of the MS medium. Most seedlings reached the top of the Petri dish within the culture time of 4 weeks. Likely due to the temperature variation between day and night, stagnant water accumulated at the bottom of many vessels being a risk for contaminations. Both *N. amplexicaulis* and *N. heliophila* exhibited robust growth across all media. For *N. amplexicaulis,* visual observation suggested that on 1/15xS_90Se roots were shorter and thicker (Fig. 7). Similarly, *M. truncatula* showed vital growth, even on MS medium supplemented with 90 µM Se. However, on 1/15xS_MS_90 Se, a clear growth inhibition and pronounced red coloration and chlorosis on the leaves were observed. In general, *N. amplexicaulis* produced the greatest biomass, while *M. truncatula* generated the least. The visual observations were confirmed by dry mass measurements of the plants (Fig. 8a). Species differed in dry mass from each other, but no significant differences were measured within species across different media (Suppl. Table S4). *N. amplexicaulis,* in particular, exhibited rapid shoot growth, resulting in the highest shoot-to-root ratio (Suppl. Figure S3).

### Selenium and S accumulation

Se concentrations varied significantly between species, treatments, and tissues, with levels being significantly higher in the hyperaccumulator *N. amplexicaulis* compared to the other species (Suppl. Table S4, Fig. 8b). On medium without Se, shoots of *N. amplexicaulis* had a concentration of Se higher than 500 µg Se g^−1^ and roots approx. 200 µg Se g^−1^. This was the only variant for *N. amplexicaulis* in which the shoot contained more Se than the root. The Se concentration in the tissue increased on media containing 90 µM Se to levels of up to 1900 µg Se g^−1^ DM as maximum concentration in the roots while the shoot contained around 1200 µg Se g^−1^ DM (Fig. 8b). In *N. heliophila*, the concentration of Se on MS_0Se was lower than 100 µg Se g^−1^ DM. The concentration of Se increased with higher Se concentration in the medium, but the accumulation was stronger in seedlings grown on media with low S. Again, the concentration was higher in the roots than shoots, reaching 500 µg Se g^−1^ DM at maximum (Fig. 8b). *M. truncatula* seedlings grown on MS_0Se and 1/15xS_0Se media contained almost no Se, indicating that the controls had no Se contamination. With increasing Se concentration in the medium the concentration increased, with higher levels in shoots than in roots. These results showed that *M. truncatula* can accumulate more than 100 µg Se g^−1^ DM without showing growth depressions, when supplied with sufficient S.

The S concentration was significantly higher in *N. amplexicaulis* compared to *M. truncatula* and *N. heliophila* (Fig. 8c). In *N. amplexicaulis*, there were trends suggesting a decrease in S concentration on 1/15xS_MS media with higher Se supplementation and an increase on full MS media. However, these trends were not statistically significant. In *M. truncatula*, S levels were similar between MS and 1/15xS_MS media. Increasing Se concentrations led to a decrease in S concentration on 1/15xS_MS, down to 1000 µg S g^−1^ DM, whereas on full MS, S concentrations increased to around 7000 µg S g^−1^ DM, similar to *N. amplexicaulis*. This increase was particularly notable in the shoots (Suppl. Figure S5).

## Discussion

### The impact of the age and genotype of seedlings

The age and treatment of legume seedlings can be very critical for regeneration with older seedlings decreasing in regeneration ability [37]. In this experiment, 5-day old *N. amplexicaulis* seedlings were used, which germinated in the dark for the first two days and displayed a morphology suitable for preparation of cotyledon, root and hypocotyl explants (Fig. 1). Using even 1 to 2-day old seedlings would have been inappropriate due to the extremely short hypocotyl and radicle. Because seedlings of legumes lose their regeneration ability in days, it may not be recommended to use older seedlings [37]. The temperature of 27 °C appeared to be suitable, not only for germination, but also during the regeneration experiments. The approached germination protocol is recommended as a practical compromise considering the strong light response of *N. amplexicaulis* seedlings resulting in extremely short hypocotyls, as it combines dark and light incubation phases. However, it cannot be confirmed if this dark–light germination timing had a biological effect on the regeneration process. Since first developmental phases of de novo shoot bud regeneration were initiated, but only at low rates, future experiments can investigate how different germination conditions influence organogenesis.

Cotyledon explants from one individual seedling were placed in one Petri dish, meaning that every replicate (= each Petri dish) was also one genetic unit. A part of the variation in callus and browning can be explained by the genetic variation within the seed batch. Therefore, the reproducible induction of shoot regeneration was remarkable, as it indicated that the regeneration capacity was not only restricted to a single genotype. The impact of the genetic background was documented in many species with only specific genotypes being competent for plant regeneration. For instance, in hemp (*Cannabis sativa*) 100 cultivars were tested and the regeneration rate of the most responsive cultivar was still only around 6% [38]. Since self-pollination is possible for *N. amplexicaulis*, inbred lines could contribute to consistency in regeneration and identification of easy to regenerate genotypes.

### Optimizing physical growth factors for tissue regeneration

An initial dark incubation period is frequently used to increase the shoot regeneration rate [39, 40] and also contributed to this protocol presumably because it reduced browning in early callus development. For tropical desert plants like *N. amplexicaulis* it could be expected that this species requires relative high temperatures also in an artificial environment [41]. A temperature of 27 °C was appropriate for callus development being already advanced after 2 weeks. Another physical factor not measured in this project but also important for the morphology and physiology of shoots is the humidity in the culture vessels. Since a less humid gas atmosphere can reduce hyperhydricity [42], other culture vessels with better gas exchange or bottom cooling systems could be tested in upcoming experiments.

### Browning could not be prevented during regeneration

The browning of explants, tissues and organs in tissue culture is a common phenomenon in plant tissue culture mainly caused by the accumulation and oxidation of phenolic compounds [43]. The factors that trigger browning are naturally high phenol concentration often formed in woody plants or in orchids [43, 44]. Commonly, browning is described as a problematic process inhibiting growth and regeneration capacity of the explants and finally leading to death of the cells [45]. Atypic for *N. amplexicaulis* was that the browning affected the tissue and callus cells internally only, while many species exude the phenols into the medium [43]. Since the first shoot induction was initiated on browned hypocotyls and roots, this browning appears not to hinder shoot regeneration in *N. amplexicaulis*. Even completely brown hypocotyl explants developed callus. However, browning became severe as soon as an explant stopped growing. On the optimized media that induced shoot formation, cotyledon explants showed significantly less browning compared to the initial attempts. Additionally, hypocotyls formed shoots and large green callus after being incubated in the dark, with a visible reduction in browning. Explants were moved into light because after 3 weeks in darkness, callus growth stopped, and browning started. In conclusion, browning processes still have to be reduced and documented critically.

### Adjusted media composition improved *N. amplexicaulis *in vitro growth

Up to now only MS medium has been used in the micropropagation protocol recently published for *N. amplexicaulis* [25]. Based on the natural habitat of *N. amplexicaulis* on rare soils with a unique natural element composition, the factors of the tissue culture conditions were varied in order to resemble more closely the natural conditions. A surprising success was obtained with medium SIM9, an MS medium with 80% of the KNO_3_ and NH_4_NO_3_ concentration and 180% of all other salts, a higher pH of 6.5 and B5 vitamins. On SIM9 supplemented with TDZ, early stages of a de novo shoot bud regeneration were observed for the first time in this species, but also an increased callus growth compared with MS and also SIM9-BAP1. An elevated ion concentration could facilitate the ion uptake through diffusion and transporters [46]. Since *N. amplexicaulis* has to deal with salinity in nature, a higher salt concentration in vitro was unlikely to cause salt stress. However, in regeneration experiment 2 it was shown that shoots could also be induced on SIM10, a MS medium with 50% of the KNO_3_ and NH_4_NO_3_ concentration. Both media had reduced N sources compared to standard MS, making the N concentration and form an objective for future medium optimization. The pH was adjusted because the natural soils of the *N. amplexicaulis* habitat are neutral to slightly alkaline [47]. A pH of 6.5 was a favorable compromise between the standard pH of 5.8 and 7 where explants did not show any growth response (data not shown).

### TDZ promoted callus growth and induced early stages of a de novo shoot regeneration

Using TDZ as cytokinin like-growth regulator was likely the breakthrough in inducing shoots initially as on SIM9-TDZ1 first organogenesis response was observed on hypocotyl and root explants. The shoot induction could be reproduced not only on SIM9-TDZ1(+ Se) but also on SIM10 proving the effect of TDZ on *N. amplexicaulis*. This result confirmed the characteristic of TDZ to be effective especially in species recalcitrant for regeneration [48]. But the disadvantages of the TDZ application like hyperhydricity and inhibition of further shoot elongation remained a challenge in the regeneration protocol, as also reported for *Albizia lebbeck* [49]*, Aloe polyphylla* [50], *Cotoneaster wilsonii* [51] and more species [52]. Incubating the freshly induced shoots for a longer time on a low TDZ concentration inhibited growth and led to a dedifferentiation and callus formation. This observation highlights the importance of properly dosing TDZ in terms of concentration and time. Likely, starting with a high concentration of 4.5 µM TDZ in the first two weeks and reducing it to 0.45 µM fulfilled the compromise of sending a strong impulse in the beginning and not disturbing the endogenous hormone balance too massively. The timing and concentration of the TDZ application should be further adjusted in future experiments.

### Root and hypocotyl explants are capable of shoot regeneration

Hypocotyl and root explants turned brown very fast and did form less callus compared to cotyledons. But explants from these organs proved their competence for regeneration although being tested in relatively low numbers only. As cotyledon explants formed callus but never regenerated shoots, even being tested in higher numbers, they can be excluded from future regeneration experiments.

Characteristic for de novo shoot primordia on hypocotyl and root explants was the deep green color of the callus from which they originated. If hypocotyls and roots showed callus, it mostly was deep green in contrast to the white-yellow callus of cotyledon explants. The color can be an indicator regeneration capacity of callus: In *Citrullus colocynthis*, a similar green color of embryogenic callus was reported [53]. The de novo regeneration from callus is classified as indirect organogenesis [22]. The rudimentary shoot buds regenerated on root explants differed in color being slightly dull indicating less hyperhydricity. Also, the spontaneously regenerated shoot, which was the only one to show further growth, appeared dull.

This spontaneous regeneration of a shoot bud on a root explant without callus induction can be defined as a direct de novo regeneration [22]. The de novo regeneration without any PGRs from a root was a rare exceptional observation induced by an individual genotype. Given that the seedlings originate from a heterogeneous population, a repetition of this experiment is required to confirm whether this event can be reproduced. This finding would be an important advantage for the future protocol development as direct organogenesis protocols are always favored due to their lower risk for somaclonal variation [54]. So far, upcoming experiments should increase the number of hypocotyl and root explants to determine the regeneration efficiency of these explant types. In this future experiment, the size of hypocotyl explants and their position and orientation should be documented, and this separately for the different genotypes represented by individual seedlings.

### Regeneration of complete shoots remains challenging

Already two weeks after shoot buds were separated from their explants and further cultivated on TDZ medium, their development stopped. Transfer to a BAP containing medium like used in the micropropagation protocol of O`Donohue et al*.* [25] stopped the development and resulted in browning. The explants were rescued and further cultivated on a TDZ containing medium with GA_3_ to promote elongation of the shoot. So far, the changes of PGRs and their concentrations did not result in proper shoot development. Currently, the primary limitation in the regeneration process is the inability to fully reprogram tissue beyond the initiation of a primordial structure, preventing its successful outgrowth and extension into a complete shoot. The failure of achieving complete shoot regeneration highlights that the recalcitrance of *N. amplexicaulis* has not been overcome yet [55]. Fundamental growth factors were already optimized and discussed to support the proper initiation of de novo shoot primordia. However, these specific conditions of the early phase may impair later developmental stages. Taking in account all tested parameters, particular the TDZ concentration and its early application were likely key factors causing physiological disturbance in later developmental stages. Future experiments could involve the use of moderate concentrations of zeatin for shoot outgrowth being a less strong natural cytokinin [56] or even PGR-free media. Additionally, the initial TDZ application should be re-evaluated broadly, with higher concentrations for very short exposure times and lower concentrations over extended periods, with particular focus on results observed after six weeks. However, given the recalcitrance and limited research in the regeneration capacity of the Mimosaceae subfamily, the developmental stages of de novo regeneration reached and established in this study represent a significant achievement.

### Effect of Se supplementation and S depletion on plant habitus

It was expected that the high concentration of 90 µM Na_2_SeO_4_ which caused growth reduction even on *N. amplexicaulis* in hydroponic system (Maggie-Anne Harvey, personal communication) had the same effect in in vitro agar-based growth systems. Reducing MgSO_4_ to 100 µM generating an almost 1:1 S:Se ratio in the medium impeded the discrimination between the elements for the plants. Obviously, the impact of Se depends strongly on the environment as 90 µM Se seemed not to be harmful in tissue culture for *N. amplexicaulis* nor the Se non-adapted species *M. truncatula*. The reason for this, could be (i) a too short growth period for rating the impact or (ii) the applied conditions were not stressful for the plants under the photomixotrophic i*n vitro* conditions. Since the plants touched the lid and filled the complete plate in 4 weeks, this system would be unsuitable for longer cultivation periods. In S starvation experiments with *A. thaliana*, the MgSO_4_ concentration for the S depleted medium varied between 0 and − 100 µM, demonstrating the S concentration used in this study represented the upper edge of an depletion experiment [57, 58]. Hyperaccumulator species show a high bioconcentration factor (shoot:substrate ratio) for ion concentrations and efficient metal sequestration, explaining why a decently S-depleted substrate is no limitation for *N. amplexicaulis* [59]. Obviously, *N. heliophila* shares this ability with *N. amplexicaulis*.

### Selenium concentration increased in tissues of all three species with increasing Se supply

The Se concentrations differed between the species and increased on Se-containing medium within each species. Differences in foliar Se concentrations were already present innately between species. The Se concentration in *N. amplexicaulis* aligned with measurements from wild populations and was expected for the seed batch used in this project, as *N. amplexicaulis* accumulates Se within its seeds. In contrast, *N. heliophila*, as an Se excluder, does not actively transfer Se into its seeds [9, 11]. The preference of *N. amplexicaulis* to take up Se explains the doubling of Se in the shoots between the MS_0Se and MS_90Se treatment and why the Se concentration increased massively in the roots on medium with Se [60]. The endogenous remobilization of Se from the relatively old leaves into the young shoot tip could explain why shoots on MS_0Se had higher Se concentrations than roots. With Se concentrations exceeding 1000 µg/g DM, *N. amplexicaulis* demonstrated its hyperaccumulation ability also under in vitro conditions [59, 61]. Surprisingly, *M. truncatula* also accumulated Se up to 300 µg Se g^−1^ DM without showing any growth depression. This is the first recorded instance suggesting that *M. truncatula* can be classified as a secondary accumulator species like its relative *Medicago sativa* [62].

### Elucidating the Se:S ratio of the species

The endogenous S concentration varied by species, with *N. amplexicaulis* showing higher S concentrations than *M. truncatula* and *N. heliophila*, potentially due to naturally elevated Se levels. Often, hyperaccumulators show a constitutively higher accumulation of other elements too [63]. For *N. amplexicaulis*, a pattern emerged where tissues with high Se concentrations also had high S concentrations, aligning with previous studies [64]. Observing that both *N. amplexicaulis* and *N. heliophila* maintained S levels on 1/15xS_MS medium even with Se supplementation suggests a high selectivity and efficient uptake system. The Se:S ratios further indicate that *N. amplexicaulis* is not experiencing stress, as this ratio can increase above one [64]. The Se:S ratio observed is characteristic for *N. amplexicaulis* and suggests an adapted selectivity for S and Se but also in *N. heliophila*. The molecular sensing and uptake mechanisms underlying this effect remain mostly unclear [65].

## Conclusions

This study successfully reported early stages of shoot regeneration from callus generated from hypocotyl and root tissues, marking the first instance of de novo shoot regeneration in a Se hyperaccumulating and the second in a Mimosaceae species. The protocol demonstrated reproducibility. The use of TDZ as a cytokinin-like growth regulator was crucial, while additional factors, such as dark incubation and an adjusted basal medium, may have also contributed to the protocol's effectiveness, although their specific impacts are not yet resolved. In an in vitro environment, *N. amplexicaulis* demonstrated hyperaccumulation of Se and the high absorption of S even though on low S supply. Extreme conditions seem to be necessary to stress *N. amplexicaulis*. This experiment also revealed for the first time that *M. truncatula* acts as a secondary Se accumulator, exhibiting behavior similar to *N. heliophila*.

## Supplementary Information

Additional file 1.

Additional file 2.

## Data Availability

The data that support this study will be shared upon reasonable request to the corresponding author.
