# Supplementary material for: Initial shoot regeneration in the selenium hyperaccumulator Neptunia amplexicaulis and in vitro test system for selenium tolerance and accumulation
Source: Nat Prod Bioprospect. 2025 Aug 4;15(1):49. doi: 10.1007/s13659-025-00532-9 (PMC12321714; doi:10.1007/s13659-025-00532-9)
Supplement: Supplementary file 1 — Additional file 1. [file 13659_2025_532_MOESM1_ESM.docx]

**SUPPLEMENTARY INFORMATION S1**

**Initial shoot regeneration in the selenium hyperaccumulator *Neptunia amplexicaulis* and *in vitro* test system for selenium tolerance and accumulation**

Bennet Buhmann^1^, Jeroen van der Woude^2^, Traud Winkelmann^1^, Antony van der Ent^2*^

^1^Institute of Plant Genetics, Section Reproduction and Development, Leibniz University Hannover, Germany.

^2^Laboratory of Genetics, Wageningen University and Research,

The Netherlands.

*Corresponding author: Antony van der Ent (antony.vanderent@wur.nl)


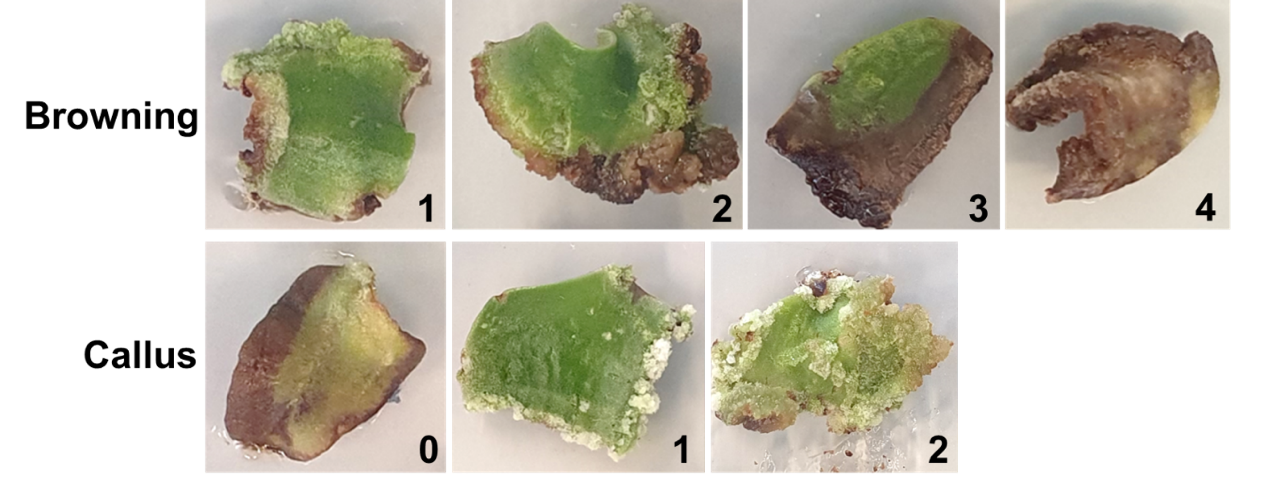


**Figure S1.** Example images for the categorization of explants regarding browning and callus formation. Images of cotyledonary explants were selected to illustrate how the visual categorization was performed. These representative images are independent examples and are not meant to show progression.


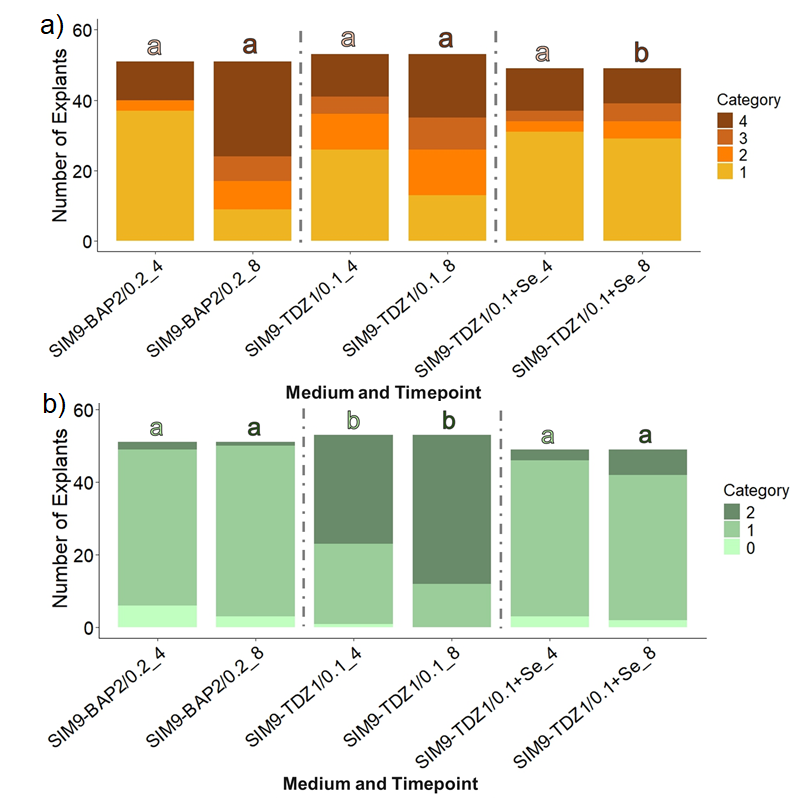


**Figure S2.** Browning of *N. amplexicaulis* explants (a) and callus formation (b) in regeneration experiment 1 according to visual rating. The absolute number of explants per treatment was recorded. The letters on the bar demonstrate significance levels (p <0.05) based on a Wilcoxon test.


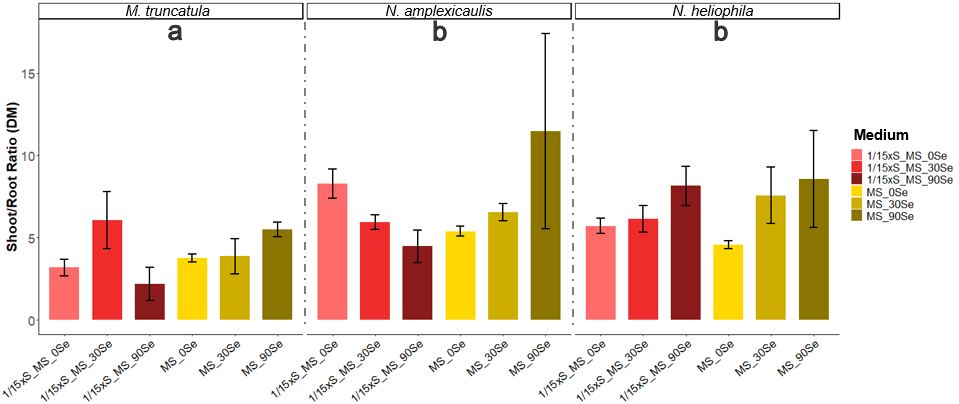


**Figure S3.** The shoot/root ratio of the dry mass depending on the plant species and medium used in the Se accumulation experiment. Given are means with standard errors of n = 6 seedlings. Statistical significance between species was determined using a post hoc Tukey test.


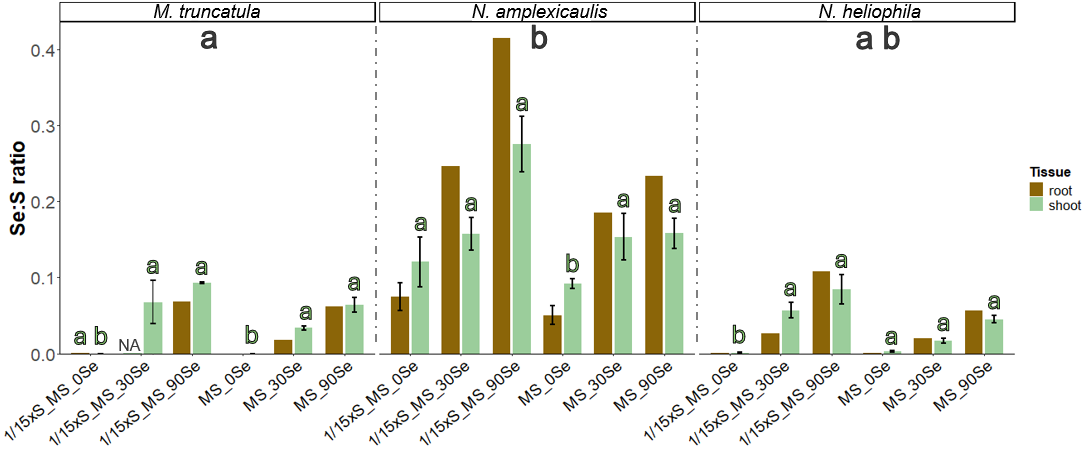


**Figure S4.** Se:S ratio in *N. amplexicaulis*, *N. heliophila* and *M. truncatula* shoot and root dry mass. The letters indicate statistically significant differences between the species and media (p <0.05) based on a pairwise Tukey test.

**Table S1.** Outcome of the Kruskal-Wallis test for the image data of regeneration experiment 1 for callus growth and browning of the explants after 4 and 8 weeks. Number of replicates and medium variants are indicated in Table 2.

|  | **Factor** | **p-values** |
| --- | --- | --- |
|  | Medium after 4 weeks | 0.0003 |
| Callus area | Medium after 8 weeks | 0.0028 |
|  | Time (4 and 8 weeks) | 0.1813 |
|  | Medium after 4 weeks | 0.9116 |
| Browned area | Medium after 8 weeks | 0.0757 |
|  | Time (4 and 8 weeks) | 0.1034 |
|  | Interaction medium and explant type | 0.6116 |

**Table S2.** Outcome of the ANOVA for regeneration experiment 2. Image data on callus formation and browning of different explants on four different media was analysed at p <0.05. Each Petri dish was considered one replicate, with n = 5-6 for cotyledons and n = 2-3 for hypocotyls and roots.

| **Category** | **Factor** | **p-value** |
| --- | --- | --- |
| Callus | Medium | 0.1085 |
|  | Explant type | 2.4*10^-5^ |
|  | Interaction medium and explant type | 0.08402 |
| Browning | Medium | 0.04207 |
|  | Explant type | 1.0*10^-10^ |
|  | Interaction medium and explant type | 0.3718 |

**Table S3.** Pairwise comparison of explant types for callus formation and browning. Mean values of the explant types were calculated by including all explants across all media. A Tukey test was conducted; n for cotyledons = 5-6, roots and hypocotlys = 2-3.

| **Category** | **Explant type** | **Mean value** | **Compared explant type** | **Mean value differences** | **p-value** |
| --- | --- | --- | --- | --- | --- |
|  | Cotyledon | 35.3 % | Cotyledon - Hypocotyl | -5.4 % | 0.3785 |
| Callus | Hypocotyl | 40.8 % | Cotyledon - Root | 17.1 % | 0.0004 |
|  | Root | 18.2 % | Hypocotyl - Root | 22.5 % | 0.0001 |
|  | Cotyledon | 23.6 % | Cotyledon - Hypocotyl | -31.7 % | 0.0001 |
| Browning | Hypocotyl | 55.3 % | Cotyledon - Root | -64.2 % | 1*10^-5^ |
|  | Root | 87.80% | Hypocotyl - Root | -32.50% | 0.0006 |

**Table S4.** Outcome of ANOVAs for the Se accumulation experiment: The effects of plant species and medium as well as their interaction (Species:Medium) were analyzed for the shoot data only. n = 6. P-values calculated from ANOVA.

| **Factor** | **Shoot dry mass** | **Se** | **S** | **S:Se ratio** |
| --- | --- | --- | --- | --- |
| Species | 2.87*10^-12^ | 2.2*10^-16^ | 5.2*10^-5^ | 0.03733 |
| Medium | 0.6852 | 3.6*10^-7^ | 0.00012 | 0.00294 |
| Species:Medium | 0.6815 | 0.00904 | 0.00209 | 0.00519 |
