# Supplementary material for: Initial shoot regeneration in the selenium hyperaccumulator Neptunia amplexicaulis and in vitro test system for selenium tolerance and accumulation
Source: Nat Prod Bioprospect. 2025 Aug 4;15(1):49. doi: 10.1007/s13659-025-00532-9 (PMC12321714; doi:10.1007/s13659-025-00532-9)
Supplement: Supplementary file 2 — Additional file 2. [file 13659_2025_532_MOESM2_ESM.docx]

**SUPPLEMENTARY INFORMATION S3**

**Initial shoot regeneration in the selenium hyperaccumulator *Neptunia amplexicaulis* and *in vitro* test system for selenium tolerance and accumulation**

Bennet Buhmann^1^, Jeroen van der Woude^2^, Traud Winkelmann^1^, Antony van der Ent^2*^

^1^Institute of Plant Genetics, Section Reproduction and Development, Leibniz University Hannover, Germany.

^2^Laboratory of Genetics, Wageningen University and Research,

The Netherlands.

*Corresponding author: Antony van der Ent (antony.vanderent@wur.nl)


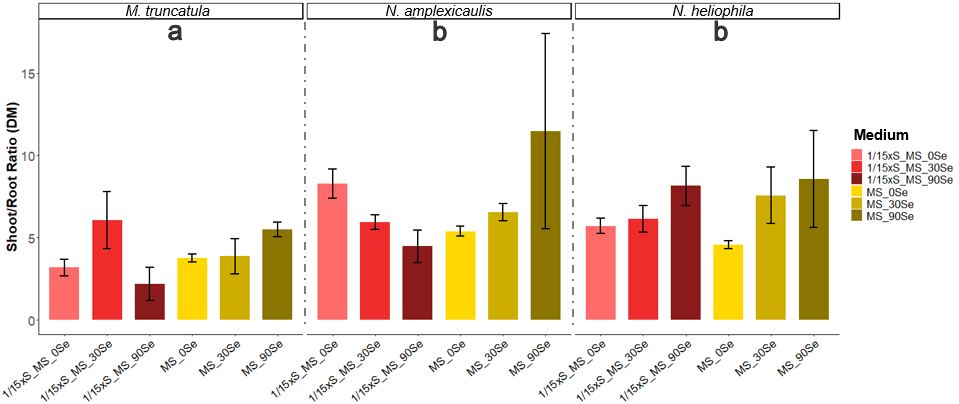


**Figure S6.** The shoot/root ratio of the dry weight for each species and medium, along with the standard error, was calculated. Statistical significance between species was determined using a post hoc Tukey test; n = 6.


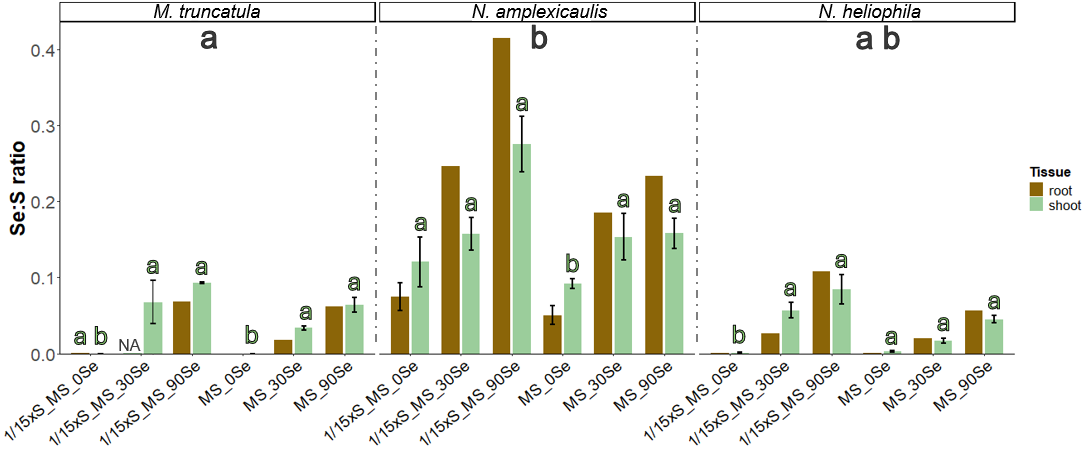


**Figure S7.** Se:S ratio of *N. amplexicaulis*, *N. heliophila* and *M. truncatula* shoot and root dry mass. The letters indicate statistically significant differences between the species (p <0.05) based on a pairwise Tukey test.
